# Rapid Synthesis and Correlative Measurements of Electrocatalytic Nickel/Iron Oxide Nanoparticles

**DOI:** 10.1038/s41598-018-22609-x

**Published:** 2018-03-15

**Authors:** Kavita M. Jeerage, Stephanie L. Candelaria, Samuel M. Stavis

**Affiliations:** 1 Applied Chemicals and Materials Division, National Institute of Standards and Technology, 325 Broadway, Boulder, Colorado, 80305 United States; 2 Center for Nanoscale Science and Technology, National Institute of Standards and Technology, 100 Bureau Drive, Gaithersburg, Maryland 20899 United States

## Abstract

Electrocatalytic core-shell nanoparticles, such as nickel/iron oxides for the oxygen evolution reaction (OER) in alkaline electrolytes, require rapid synthesis and measurement for practical use. To meet this challenge, we investigated a novel process of adding Ni(II) species to Fe nanoparticles immediately after synthesis, which we expected to yield Ni-rich shells around Fe-rich cores. Cyclic voltammetry showed that the overpotential decreased as the molar ratio of Ni to Fe in the synthesis vessel increased from 0.2 mol Ni:1 mol Fe to 1.5 mol Ni:1 mol Fe, consistent with an increase of Ni composition. Unexpectedly, the overpotential increased abruptly at 2.0 mol Ni:1 mol Fe. X-ray photoelectron spectroscopy revealed that this synthesis ratio resulted in less Ni at the nanoparticle surfaces than lower synthesis ratios. These results demonstrate the sensitivity of rapid electrochemical measurements to surface composition, and the limits of Ni(II) adsorption and reduction to rapidly form Ni-rich shells around Fe-rich cores. Cyclic voltammetry also showed that the onset of the methanol oxidation reaction (MOR) correlates with the oxidation of Ni(OH)_2_ to NiOOH. Therefore, tuning materials to improve performance as OER catalysts also improves their performance as MOR catalysts.

## Introduction

Engineered nanoparticles with multiple functions from core-shell structures have applications ranging from medicine to catalysis. For example, nanoparticles consisting of earth-abundant cores within noble-metal shells provide magnetic functionality and biological compatibility for magnetic resonance imaging^1,2^ and catalytic activity for methanol oxidation^3^. However, the preparation and characterization of such nanoparticles is challenging, for several reasons. First, the scarcity of noble metals motivates the development of synthetic processes to reduce shell thickness or replace noble metals with more economical materials. Next, any modification of a nanoparticle core, such as by addition of a shell material or covalent linkage to a polymer, can yield heterogeneous surface structures and functions. Heterogeneous products can result even from the synthesis of small batches of nanoparticles under precise control in a laboratory environment. The rapid synthesis of large batches of nanoparticles in a manufacturing environment presents further challenges. Last, characterization of nanoparticle surface structures and functions with high throughput at low cost for industrial application is currently difficult.

New techniques are emerging to rapidly and economically evaluate the structure and function of core-shell nanoparticles. Recently, Tschulik *et al*. distinguished intact from broken shells for engineered nanoparticles with a core-shell morphology^4^. Their elegant method probes the nanoparticle core and shell independently, by reductive dissolution of imperfectly protected cores and oxidative dissolution of shells, at separate potentials. Interestingly, their evaluation of a model system consisting of a magnetite core and gold shell revealed that less than 1% of the nanoparticles had an intact shell that was sufficient to protect the core from dissolution. Tschulik *et al*. observed that transmission electron microscopy (TEM) can also provide information on imperfectly protected cores, but only for a relatively small sample, whereas electrochemical measurements readily sample many nanoparticles in an ensemble.

For electrocatalytic nanoparticles, X-ray photoelectron spectroscopy (XPS) is the most common characterization method, aside from electrochemical measurements. XPS probes a depth of approximately 10 nm and can identify surface constituents, including contaminants and oxide/hydroxide species, but is not suitable for routine analysis due to low throughput and high cost. We hypothesize that electrochemical measurements, which are relatively rapid and inexpensive, can be correlated with a more detailed evaluation of nanoparticle surface species such as by XPS. As a model system to test this hypothesis and simultaneously investigate a novel process to rapidly synthesize electrocatalytic nanoparticles, we study Fe(0) cores with Ni-rich shells. Recent studies of the surface composition and electrochemical response of Ni/Fe hydroxides or oxyhydroxides motivate our study of this system. Ni/Fe hydroxides or oxyhydroxides can be formed by cathodic deposition^5^ and perform well as electrocatalysts^6^ for the oxygen evolution reaction (OER) in alkaline electrolytes: 4OH^-^ → 2H_2_O + O_2_(g) + 4e^-^. Amorphous films formed by annealing^7,8^ or photochemical decomposition^9^ of a precursor film have allowed precise control of the metal stoichiometry. These studies have demonstrated that the presence of Fe in the Ni films strongly influences the voltammetric features at potentials lower than the OER onset potential. While films with 0% Fe or 100% Fe have poor performance, there is a large range of intermediate compositions from approximately 15% Fe to approximately 50% Fe with good performance^10^. The oxidation peak that appears at potentials just below the OER onset potential is the oxidation of nickel(II) hydroxide to nickel(III) oxyhydroxide by the reaction: Ni(OH)_2_ + OH^-^ ↔ NiOOH + H_2_O + e^−^. Previous studies have generally concluded that NiOOH is the catalytically active material^6^.

Fe(0) nanoparticles can be formed by reducing Fe(III) with sodium borohydride in aqueous solution under ambient conditions^11^. XPS shows that, immediately after synthesis, nanoparticles have a surface layer of iron(III) oxyhydroxide, formed by the reaction: Fe(s) + 2H_2_O → FeOOH + 1.5H_2_(g). Batch contact experiments with aqueous Ni(II) indicate both adsorption and reduction, with full reduction of Ni(II) after several hours. Ni(II) can displace Fe(0) because Fe(0) is a good electron donor (E° = −0.447 V *vs*. SHE^12^) and Ni(II) can accept electrons (E° = −0.257 V *vs*. SHE^12^), where E° is the standard reduction potential and SHE is the standard hydrogen electrode. The FeOOH shell allows rapid adsorption of Ni(II) on the surface, while the ZVI core provides electrons to reduce Ni(II)^13^. This phenomenon can also be utilized in reverse. Recent work showed that the immersion of Ni foam electrodes in a Fe(NO_3_)_3_ solution oxidized them by the reaction NO_3_^−^ + 2 H^+^  + 2e^−^ → NO_2_^−^ + H_2_O^14^. This process simultaneously released Ni(II) and increased the pH of the Fe(NO_3_)_3_ solution near the surface of the Ni, causing amorphous Ni/Fe hydroxides to precipitate onto the Ni foam electrode. In this synthetic process, Fe(NO_3_)_3_ concentration and Ni immersion time are the independent variables. Here, we investigate the possibility of controlling the quantity of Ni species on a Fe/FeOOH nanoparticle surface by varying the molar ratio of Ni to Fe in the synthesis vessel, allowing 30 min for complete Fe reduction by sodium borohydride and 15 min for Ni(II) adsorption. Following nanoparticle isolation, we measured the nanoparticles by cyclic voltammetry in 1 mol/L NaOH, cycling them above the OER onset potential. We evaluated the bulk composition of the nanoparticles by inductively coupled plasma atomic emission spectroscopy (ICP-AES) and evaluated their surface composition by XPS.

We find that increasing the molar ratio of Ni to Fe in the synthesis vessel provides limited control over the quantity of Ni species on the Fe/FeOOH surface. The quantity of Ni incorporated initially increases with the molar ratio of Ni to Fe in the synthesis vessel, but levels off and then, unexpectedly, decreases. From cyclic voltammetry in an alkaline electrolyte, the position of the pre-catalytic oxidation peak corresponding to the Ni(OH)_2_ to NiOOH transition and the OER overpotential at 10 mA/cm^2^, a common metric for comparison^15^, clearly indicates changes in the surface composition, which XPS verifies. These results demonstrate correlation of electrochemical and spectroscopic measurements, and provide information about the limits of Ni(II) adsorption and reduction as a process to rapidly synthesize Ni-rich shells around Fe-rich cores. Cyclic voltammetry also reveals that the methanol oxidation reaction (MOR) coincides with the Ni(OH)_2_ to NiOOH transition. Therefore, tuning materials to improve their performance for OER catalysis improves them for MOR catalysis.

## Results and Discussion

### Oxygen Evolution Reaction (OER) and Methanol Oxidation Reaction (MOR) vs. Bulk Composition

**Figure 1:**
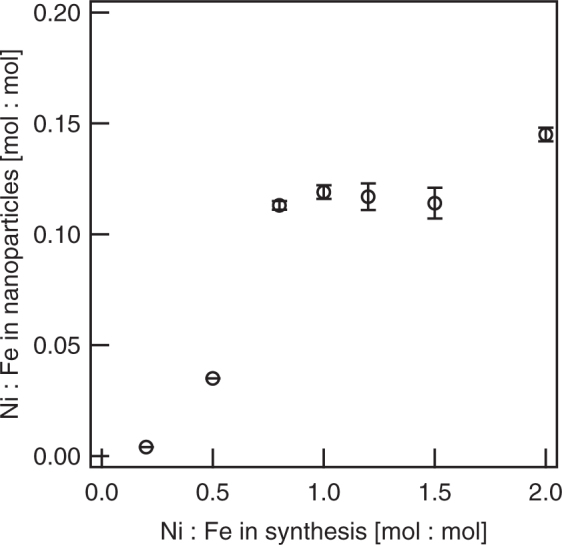
Molar ratio of Ni:Fe from bulk composition analysis. Nanoparticles from the synthesis ratios 0.2 mol Ni:1 mol Fe, 0.5 mol Ni:1 mol Fe, and 2 mol Ni:1 mol Fe were statistically different from all other synthesis ratios (*p* < 0.001). Nanoparticles from the synthesis ratios 0.8 mol Ni:1 mol Fe, 1 mol Ni:1 mol Fe, 1.2 mol Ni:1 mol Fe, and 1.5 mol Ni:1 mol Fe were not statistically different from each other (*p* > 0.05). Vertical bars indicate standard deviations and are smaller than the data markers in several instances.

We determined the bulk composition of the synthesized nanoparticles by ICP-AES. Based on complete reduction of Fe(II) by NaBH_4_, Ni(II) reduction accompanied by Fe oxidation, and the similarity in the atomic weights of Fe of 55.8 g/mol and Ni of 58.7 g/mol, we expected a total metal content in the suspension of 2 g/L. The metal content for all batches of nanoparticles was 2.0 g/L ± 0.2 g/L and none of the batches showed a significant statistical difference from the overall mean. We report measured quantities with uncertainties as mean values ± standard deviations. Based on measurements of Ni and Fe content, we calculated the molar ratio of Ni:Fe for each batch of nanoparticles (Fig. 1). Bulk composition analysis suggests that the amount of Ni(II) added to the synthesis vessel limited adsorption of Ni(II) by FeOOH and/or the reduction of Ni(II) by the Fe core for synthesis ratios up to 0.8 mol Ni:1 mol Fe. Synthesis ratios from 0.8 mol Ni:1 mol Fe to 1.5 mol Ni:1 mol Fe were indistinguishable by bulk composition analysis, but the final synthesis ratio (2.0 mol Ni:1 mol Fe) had a larger amount of Ni. Higher Ni:Fe ratios in the nanoparticles can indicate more Ni in the shell from more Ni(II) adsorption and possibly Ni(II) reduction, or the formation of Ni-rich organic nanoparticles, which a previous study observed by scanning transmission electron microscopy (STEM) of nanoparticle batches formed with 1.0 mol Ni:1 mol Fe in the synthesis vessel^16^.

**Figure 2:**
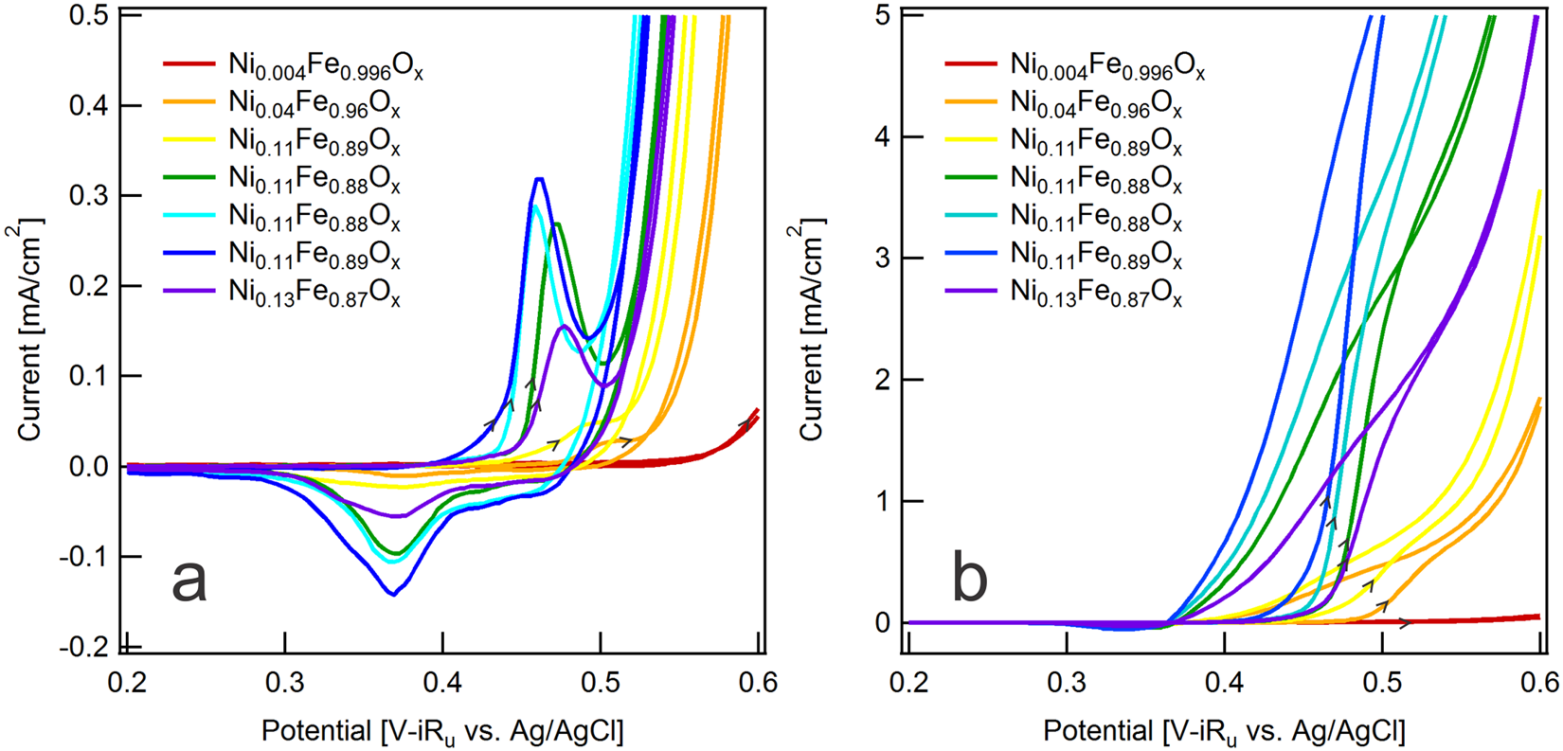
Cyclic voltammograms showing (**a**) steady-state O_2_ evolution (30^th^ cycle) and (**b**) steady-state CH_3_OH oxidation (10^th^ cycle) at 20 mV/s for all synthesis ratios and resulting bulk compositions of nanoparticles. We calculate current density from the geometric area of the electrode.

We can readily discern the effect of adding Ni species to the Fe/FeOOH nanoparticles by examining the steady-state oxygen evolution reaction (OER) by cyclic voltammetry (Fig. 2a). For the most Fe-rich nanoparticles with a composition of Ni_0.004_Fe_0.996_O_x_, oxygen evolution remained kinetically challenging and the overpotential for a current density of 10 mA/cm^2^ was 590 mV. The next nanoparticle composition of Ni_0.04_Fe_0.96_O_x_ had an order of magnitude more Ni and a correspondingly lower overpotential of 470 mV. Although the next four batches of nanoparticles had indistinguishable bulk compositions of Ni_0.11_Fe_0.89_O_x_, differences between them are evident in the cyclic voltammograms, and the overpotentials decreased from 440 mV to 405 mV. Finally, for the most Ni-rich nanoparticles with a composition of Ni_0.13_Fe_0.87_O_x_, the overpotential increased to 430 mV.

There are several sources of measurement uncertainty for these overpotential values. First, the potential of the Ag/AgCl reference electrode changes in alkaline electrolytes due to Ag_2_O formation. Second, the pH of the NaOH electrolyte might deviate slightly from 14, which is challenging to measure with a glass electrode. We separated the Ag/AgCl reference electrode from the alkaline electrolyte with a salt bridge, verified its potential against a reference, and used the same batch of 1 mol/L NaOH electrolyte for all experiments. Therefore, while the absolute overpotentials have an uncertainty of 12 mV, the relative overpotentials have an uncertainty of approximately 1 mV. Last, any variability of the mass and surface area of nanoparticles on the electrode contributes additional uncertainty to the absolute overpotentials that we do not quantify, but the corresponding shift in the pre-catalytic oxidation peak provides confidence in the relative overpotentials.

The cyclic voltammograms for Ni_0.004_Fe_0.996_O_x_ did not have a pre-catalytic oxidation peak, which corresponds to oxidation that occurs at potentials lower than the potential required for catalytic oxygen evolution. This is characteristic of Fe(III) sites and suggests that the shell composition was FeOOH, which is consistent with the synthesis process^11^. The other cyclic voltammograms did have a pre-catalytic oxidation peak, although it might have been obscured partially by OER catalysis. Previous studies have attributed the pre-catalytic oxidation peak to the oxidation of Ni(OH)_2_ to NiOOH, which is active for OER catalysis^7^. As the bulk composition changed from Ni_0.04_Fe_0.96_O_x_ to slightly more Ni-rich compositions, the precatalytic oxidation peak shifted to lower potentials. However, this peak shifted to a higher potential for Ni_0.13_Fe_0.87_O_x_. The broad reduction peak at approximately 0.37 V, which we attribute to the reduction of NiOOH to Ni(OH)_2_, did not shift in potential. We synthesized additional batches of nanoparticles using the same process. Again, nanoparticles formed with 2.0 mol Ni:1 mol Fe had a higher overpotential for OER catalysis than nanoparticles formed with less Ni(II) in the synthesis vessel. The cyclic voltammograms in Fig. 2a are surprising because previous studies of Ni/Fe (oxy)hydroxides showed that the pre-catalytic oxidation peak was not observable for thin-film compositions with 40% Fe or more^9,10^. This discrepancy suggests that bulk composition analysis does not adequately describe the nanoparticle surface, which probably contains more Ni and less Fe. Interestingly, the cyclic voltammograms for the four nanoparticles with identical bulk composition showed a notable shift in the potential of the pre-catalytic oxidation peak, again suggesting that bulk composition analysis does not adequately describe the nanoparticle surface.

The cyclic voltammograms changed dramatically following the addition of 1 mol/L CH_3_OH to the 1 mol/L NaOH electrolyte (Fig. 2b). The oxidation of Ni(OH)_2_ to NiOOH is no longer visible as a pre-catalytic oxidation peak. Instead, during the forward scan and at approximately the same potential, the current increased. We attribute this current to a catalytic methanol oxidation reaction (MOR)^16^. Similar to OER catalysis, the onset of MOR catalysis shifted to lower potentials as the bulk composition changed from Ni_0.004_Fe_0.96_O_x_ to Ni_0.04_Fe_0.96_O_x_ to slightly more Ni-rich compositions, but shifted to a higher potential for Ni_0.13_Fe_0.87_O_x_. Therefore, by shifting the potential at which Ni(OH)_2_ oxidizes to NiOOH, additional Ni within the Fe/FeOOH nanoparticle shell impacts methanol oxidation. During the reverse scan, methanol oxidation proceeded until the potential where all NiOOH would be reduced to Ni(OH)_2_.

**Figure 3:**
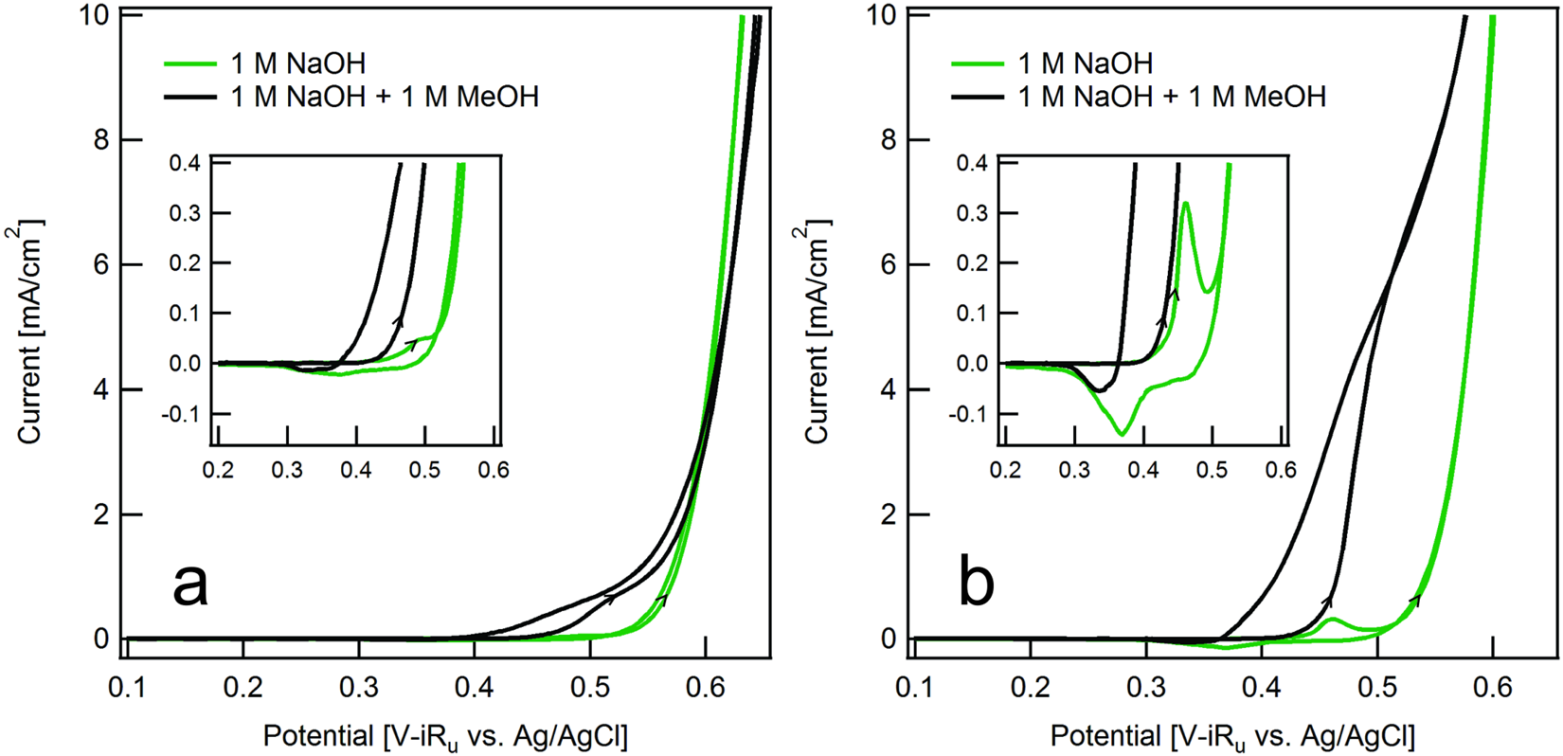
Cyclic voltammograms directly comparing steady state O_2_ evolution (30^th^ cycle) and steady state CH_3_OH oxidation (10^th^ cycle) at 20 mV/s for synthesis ratios of (**a**) 0.8 mol Ni:1 mol Fe and (**b**) 1.5 mol Ni:1 mol Fe. We calculate current density from the geometric area of the electrode. Insets show an expanded current scale.

Direct comparison of the OER and MOR voltammograms reveals interesting features. For the 0.8 mol Ni:1 mol Fe synthesis ratio (Fig. 3a), the pre-catalytic oxidation peak in the OER catalysis did not reach a current of 0.1 mA/cm^2^ and is therefore only visible at the expanded current scale. Furthermore, OER catalysis partially obscured this peak. In contrast, for the 1.5 mol Ni:1 mol Fe synthesis ratio (Fig. 3b), the pre-catalytic oxidation peak in the OER catalysis reached approximately 0.3 mA/cm^2^ and is visible at both current scales. Due to the shift of the pre-catalytic peak to a lower potential, OER catalysis did not obscure this peak. These observations suggest an increase in the Ni:Fe ratio at the nanoparticle surface. At the expanded current scale, it is apparent that the MOR current increases at approximately the same potential at which Ni(OH)_2_ oxidizes to NiOOH, suggesting that NiOOH is the catalytically active material for both reactions. Previous work with Ni foam electrodes also indicated that NiOOH is the catalytically active material for MOR catalysis^17^ and observed masking of the Ni(OH)_2_/NiOOH transition. However, previous studies have not reported the correspondence between MOR catalysis and the Ni(OH)_2_/NiOOH transition for the Fe-rich Ni/Fe oxides that we studied here. Our observation indicates that Ni/Fe oxides with optimal compositions for OER catalysis will perform well for MOR catalysis.

### X-ray Photoelectron Spectroscopy (XPS) Surface Analysis

XPS of nanoparticles provides information about shell structure, probing the surface composition to a depth of approximately 10 nm without additional sample preparation, such as ion sputtering. We used XPS survey spectra with an energy resolution of 1 eV in conjunction with empirical sensitivity factors to estimate the atomic concentration of elements present at the nanoparticle surfaces (Table 1). Atomic concentrations from XPS measurements can have errors of up to 10%^18^ in part because the analysis assumes that the probed volume is homogeneous, which is improbable. While this limits their accuracy, XPS measurements are nonetheless precise and useful to compare variable surface compositions of samples with similar bulk compositions, such as the ones that we have investigated here. Table 1 shows that the samples had approximately 50% oxygen, which indicates that the metals were present as oxides or oxyhydroxides. In addition, the samples had approximately 20% to 30% carbon, due to adventitious carbon and the ligand stabilizers. For the metals, the most reasonable quantity to compare is the Ni:Fe ratio at the surface. Survey scans confirmed that the 0.2 mol Ni:1 mol Fe and 0.5 mol Ni:1 mol Fe synthesis ratios resulted in very little Ni incorporation by the Fe/FeOOH nanoparticles. Furthermore, the quantity of Ni that we measured in these nanoparticles was near the limit of detection of the XPS measurement. From the 0.8 mol Ni:1 mol Fe synthesis ratio to the 1.5 mol:1 mol Fe synthesis ratio, there was an increase in the Ni:Fe ratio at the surface, which bulk composition analysis did not show. For the 2.0 mol Ni:1 mol Fe synthesis ratio, there was a distinct decrease in the Ni:Fe ratio at the surface. Taken together, these results indicate that modulating the Ni:Fe ratio in the synthesis vessel does not monotonically control the Ni:Fe ratio at the nanoparticle surface. Interestingly, in previous work in which the immersion of Ni foam electrodes in an Fe(NO_3_)_3_ solution led to the formation of Ni/Fe hydroxides^14^, high concentrations of Fe(NO_3_)_3_, which are analogous to the Ni:Fe ratio in our synthetic approach, caused the Ni/Fe hydroxide layers to detach from the bulk electrode, creating an inferior catalyst to those produced from low concentrations of Fe(NO_3_)_3_. Furthermore, the surface compositions that we synthesized and characterized did not reach the quantity of Ni reported to produce the lowest overpotentials for OER catalysis. Recent studies have reported 60% Ni^10^, 80% Ni^9^, and 90% Ni^7^ as the best compositions for thin Ni/Fe oxide films.

**Table 1:** Relative elemental composition from surface analysis to a depth of approximately 10 nm.

Synthesis Ratio [mol Ni: mol Fe]	Ni [%]	Fe [%]	O [%]	C [%]	Ni:Fe [ ]
0.2:1.0	2 × 10^−1^	2 × 10^1^	5 × 10^1^	3 × 10^1^	≤0.01
0.5:1.0	1 × 10^−1^	3 × 10^1^	5 × 10^1^	2 × 10^1^	≤0.01
0.8:1.0	2 × 10°	2 × 10^1^	5 × 10^1^	3 × 10^1^	0.1
1.0:1.0	3 × 10°	2 × 10^1^	5 × 10^1^	2 × 10^1^	0.2
1.2:1.0	2 × 10°	1 × 10^1^	5 × 10^1^	3 × 10^1^	0.2
1.5:1.0	4 × 10°	1 × 10^1^	5 × 10^1^	3 × 10^1^	0.4
2.0:1.0	2 × 10°	2 × 10^1^	5 × 10^1^	2 × 10^1^	0.1

We sampled and averaged two spots for synthesis ratios from 0.8 mol Ni:1 mol Fe to 2.0 mol Ni:1 mol Fe.

XPS survey spectra also detected multiple impurities at the nanoparticle surface. However, none of these were elements such as Ir or Ru^19^ which we would expect to affect electrocatalytic activity. Phosphorus was present at all sampling spots, 12 in total, with a concentration of 1.7% ± 0.7%. Nitrogen was present at all sampling spots, with a concentration of 1.6% ± 0.4%. Phosphorus resulted from the ATMP stabilizer, whereas nitrogen resulted primarily from the PVP_40_ stabilizer. Both stabilizers contributed to the carbon and oxygen content, as well, although the primary source of carbon is adventitious carbon and the primary source of oxygen is metal oxides. Chlorine was present at all sampling spots, with a concentration of 0.6% ± 0.2%, from the nickel chloride solution, even when the quantity of nickel was near the detection limit. Sulfur was present at half the sampling spots, with a concentration of 0.2% ± 0.1%, from the iron sulfate solution.

Surface analysis provides additional information for interpreting the trends in cyclic voltammograms of OER catalysis (Fig. 2a). The four synthesis ratios that yielded identical bulk compositions (0.8 mol Ni:1 mol Fe to 1.5 mol Ni:1 mol Ni) had surface compositions that increased from Ni_0.1_Fe_0.9_O_x_ to Ni_0.3_Fe_O.7_O_x_. The synthesis ratio that yielded surprisingly poor performance for OER catalysis despite the highest bulk composition (2.0 mol Ni:1 mol Fe) had a surface composition of Ni_0.1_Fe_0.9_O_x_. The 2.0 mol Ni:1 mol Fe and 0.8 mol Ni:1 mol Fe synthesis ratios had similar overpotentials, 440 mV and 430 mV, respectively. Previous studies have reported optimum compositions of 10% Fe^7^, 20% Fe^9^, and 40% Fe^10^, but this apparent discrepancy is explainable by measurements that indicate thin-film compositions between 15% Fe and 50% Fe, have an overpotential that varies by at most 20 mV^10^. The high overpotentials that we observed here are consistent with electrodeposited catalysts having 70% or more Fe. At a specific overpotential of 350 mV, the current density increased by more than two orders of magnitude as the nanoparticle shell increased to approximately 30% Ni. Although we have probed only a small fraction of the Ni/Fe composition space, these trends are consistent with those of previous studies.

**Figure 4:**
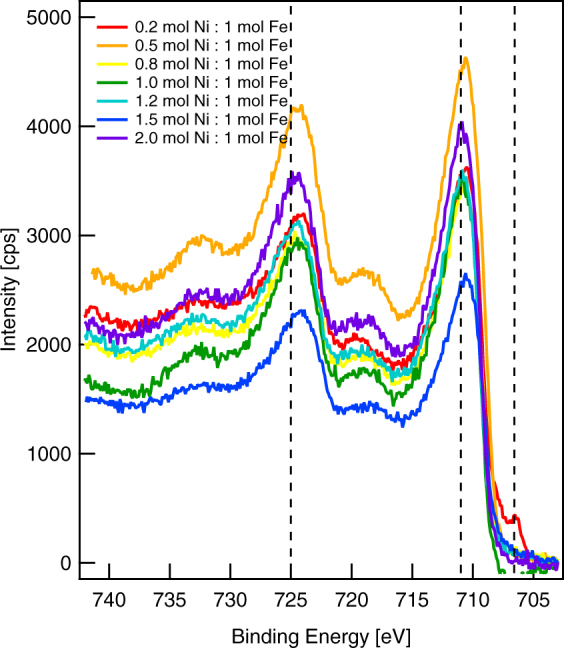
X-ray photoelectron spectra showing the Fe 2p region. Dashed lines indicate binding energies for Fe(III) (725.0 eV and 711.0 eV) and Fe(0) (706.5 eV). To align the spectra, we set the intensity at a binding energy of 703.0 eV to 0. We show one representative spectrum per synthesis condition.

We obtained XPS spectra with an energy resolution of 0.1 eV to examine Fe oxide and Ni oxide species at the surface. Such spectra can sometimes identify the oxide species at a surface based solely on the binding energy of the 2p_3/2_ signal by assuming a single peak maximum, but this approach is not valid for many transition metals, including Fe and Ni^20^. High-resolution spectra of the Fe 2p region (Fig. 4) show that only the 0.2 mol Ni:1 mol Fe synthesis ratio resulted in a thin enough shell for metallic, zero-valent iron from the core to be evident at 706.5 eV. Fe_2_O_3_ and FeOOH both have a broad peak at 711.0 eV due to Fe(III)^21^. Fe_3_O_4_, which can also be written as FeO·Fe_2_O_3_ and has an Fe(II)/Fe(III) ratio of 1:2, does not have a satellite peak above the Fe 2p_3/2_ peak at 711.0 eV^22^. Therefore, we do not consider it further. One way to distinguish between Fe_2_O_3_ and FeOOH is to examine the O 1 s spectra, which contain three distinct peaks: the peak at 529.6 eV corresponding to O^2−^, the peak at 531.0 eV corresponding to OH^−^, and the peak at 532.2 eV corresponding to adsorbed H_2_O. For FeOOH, the OH^−^/O^2−^ ratio should be approximately one, corresponding to the stoichiometry and reported values, whereas for Fe_2_O_3_, there should be a single peak at 529.6 eV. Examining the O 1 s spectrum for the 0.2 mol Ni:1 mol Fe synthesis ratio, which contained almost no Ni, we observe two distinct peaks. We compared peak areas after Shirley-background subtraction^23^ to determine the OH^−^/O^2−^ ratio, which is 0.9, indicating FeOOH as we expected.

**Figure 5:**
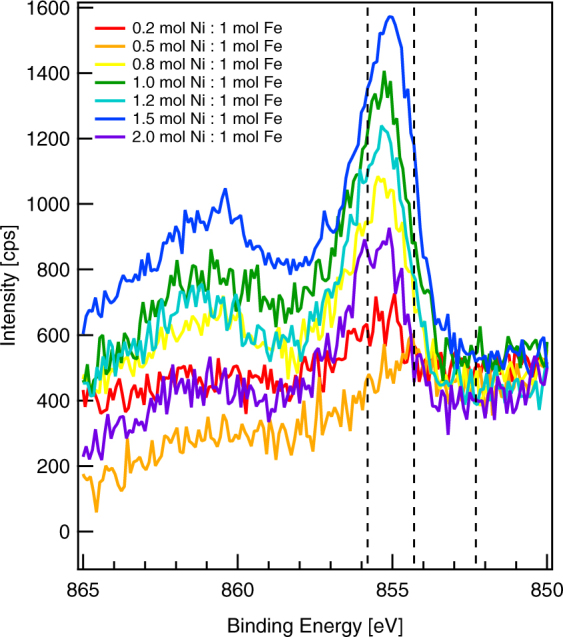
X-ray photoelectron spectra showing the Ni 2p region. Dashed lines indicate binding energies for Ni(III) (855.8 eV), Ni(II) (854.3 eV), and Ni(0) (852.3 eV). To align the spectra, we set the intensity at a binding energy of 850.0 eV to 500. We show one representative spectrum per synthesis condition.

High-resolution spectra of the Ni 2p region (Fig. 5) are similarly challenging to interpret. Metallic nickel has a main peak at 852.3 eV with two satellite peaks above this binding energy^24^. We did not find any evidence for Ni(0) species despite the synthetic process, which involves adsorption of Ni(II) species and possible reduction by Fe(0). There are a few possible explanations for this. It is possible that the FeOOH shell prevents interaction and electron transfer between Ni(II) and Fe(0) in the nanoparticle core. It is also possible that Ni(II) reduction in the synthesis vessel was followed by oxidation in the atmosphere during nanoparticle isolation and during drop-casting onto a solid surface, which we did not perform under anaerobic conditions. We conclude that any Ni in the nanoparticle shell already consists of one or more Ni oxides or oxyhydroxides.

Binding energies for Ni(II) and Ni(III) species are 854.3 eV and 855.8 eV, respectively^24^, but broad peaks make any assignment challenging. NiO and Ni(OH)_2_ are possible Ni(II) species and NiOOH is a possible Ni(III) species. In the O 1 s region, we expect that NiO would have a peak at 531.0 eV (OH^−^) that comprises approximately 30% of the area and a peak at 529.6 eV (O^2−^), whereas we expect that Ni(OH)_2_ would have a single peak at 531.0 eV (OH^−^). Similar to FeOOH, we expect that NiOOH would have O 1 s peaks at 531.0 eV (OH^−^) and 529.6 eV (O^2−^) of equal areas. Due to the surprisingly low quantity of Ni at the surface for most of these samples, we can draw only limited conclusions about Ni species. We note that as the ratio of Ni:Fe at the surface increased, the area of the peak at 531.0 eV (OH^−^) also increased, leading to an increase in the OH^−^/O^2−^ ratio to approximately 1.6 for the 1.5 mol Ni:1 mol Fe synthesis ratio. This suggests that Ni may be primarily Ni(OH)_2_, but does not rule out the possibility of NiOOH species.

**Figure 6:**
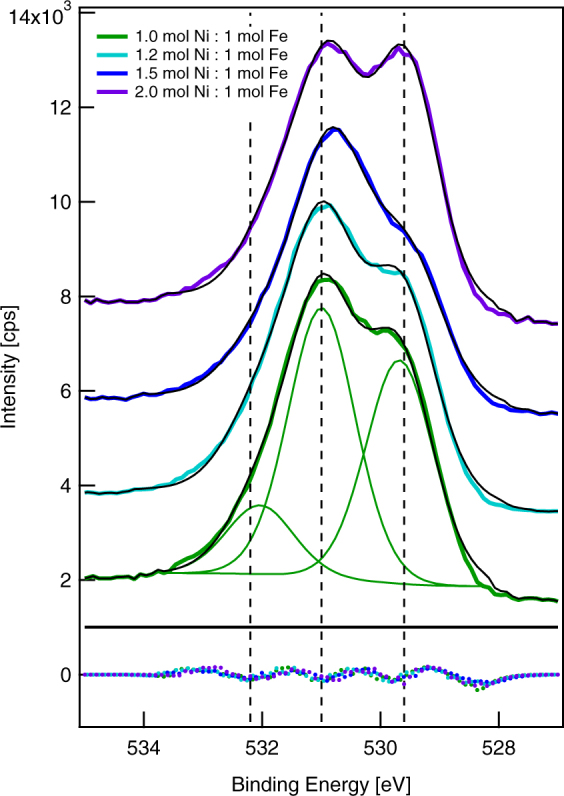
X-ray photoelectron spectra showing the O 1 s region. Dashed lines indicate binding energies for H_2_O (532.2 eV), OH^−^ (531.0 eV), and O^2−^ (529.6 eV). To separate the spectra, we offset the intensity at a binding energy of 525.0 eV by 2000 cps. Solid green lines show decomposed peaks for the 1.0 mol Ni:1 mol Fe synthesis ratio and solid black lines show the sum of the decomposed peaks for all synthesis conditions. Dotted lines at the bottom of the figure show the residuals between the measured spectra and the summed peaks.

Due to this uncertainty, high-resolution spectra of the O 1 s region (Fig. 6) provide valuable information. We decomposed each spectrum^18^ into three peaks centered at approximately 532.2 eV (H_2_O), 531.0 eV (OH^−^), and 529.6 eV (O^2−^), using a Gaussian/Lorentzian product formula with a mixing parameter of 0.3, indicating that the peaks are predominantly Gaussian in character. Figure 6 shows the decomposed peaks for one spectrum as well as the summed peaks for all spectra, which match the original spectra well, based on the residuals. We used the areas of the decomposed peaks to calculate the ratio of the OH^−^ peak to the O^2−^ peak. This ratio increased as the amount of Ni at the surface increased. For the 1.5 mol Ni:1 mol Fe synthesis ratio, the O^2−^ peak has become a shoulder and the ratio is approximately 1.6, suggesting a higher fraction of Ni(OH)_2_. For the 2.0 mol Ni:1 mol Fe synthesis ratio, the O^2−^ peak abruptly increases and the ratio is approximately 1.0, which is similar to the 0.8 mol Ni:1 mol Fe synthesis ratio. By contrast, the fraction of oxygen associated with water remains constant from approximately 15% to 20% of the total and does not show any trends with respect to the Ni:Fe ratio in the synthesis vessel. Although Fig. 6 shows only one spectrum for each synthesis condition, we sampled two spots to test sample heterogeneity. The sample from the 2.0 mol Ni:1 mol Fe synthesis ratio was the only one in which the spectra did not match. The mismatch between the bulk composition and the surface analysis for nanoparticles from the 2.0 mol Ni:1 mol Fe synthesis ratio suggests the formation of Ni-rich organic nanoparticles formed from Ni(II) and the ATMP stabilizer^16^. By probing the composition of individual nanoparticles or small clusters of nanoparticles, STEM in combination with energy dispersive X-ray spectroscopy (EDS) demonstrates that our synthesis does yield Ni-rich organic nanoparticles, as well as Ni/Fe nanoparticles with the core-shell structure that we expected. We provide examples of both compositions in the Supplementary Information. Whereas STEM provides high-resolution images of single nanoparticles, electrochemical measurements provide ensemble characterization of nanoparticle surface structures with high throughput and low cost.

## Conclusions

We have investigated the relationship between synthesis conditions, material composition, and electrochemical performance of Ni/Fe nanoparticles with a core–shell structure. Bulk composition analysis showed that increasing the quantity of Ni(II) species added to Fe(0) nanoparticles immediately after synthesis increased the molar ratio of Ni:Fe in the nanoparticles; however, the increase was not monotonic. Synthesis ratios from 0.8 mol Ni:1 mol Fe to 1.5 mol Ni:1 mol Fe yielded identical bulk composition, but cyclic voltammetry demonstrated that both the onset of oxygen evolution (OER) and the onset of methanol oxidation (MOR) varied for these nanoparticles. Electrochemical measurement results correlated with the composition of the nanoparticle surface, in particular, an increase in Ni(OH)_2_ at the surface. We expected the 2.0 mol Ni:1 mol Fe synthesis ratio to yield nanoparticles with the best electrochemical performance based on their bulk composition, but cyclic voltammetry instead showed a performance decrease. Again, electrochemical measurements correlated with the composition of the nanoparticle surface, which was primarily FeOOH rather than Ni(OH)_2_. We conclude that our novel synthesis process, based on adsorption and reduction of Ni(II) species, has an upper limit to its ability to rapidly form Ni-rich shells around Fe cores. A possible explanation for this limit is that high concentrations of Ni(II) in the synthesis vessel promote the formation of Ni-rich organic nanoparticles, leaving less Ni(II) available for adsorption and reduction. Further measurements would clarify this possibility, while maintaining a low Ni(II) concentration in the synthesis vessel and increasing the time allowed for Ni(II) adsorption could potentially address this issue, but decrease synthesis throughput. We conclude that electrochemical measurements can rapidly identify nanoparticles with surface compositions having a greater proportion of Ni(OH)_2_ in comparison to FeOOH, which we identified by high-resolution X-ray photoelectron spectra. We also conclude that for Ni/Fe oxides with Fe-rich compositions, CH_3_OH oxidation correlates with a pre-catalytic peak seen during O_2_ evolution that is attributed to the oxidation of Ni(OH)_2_ into NiOOH. Therefore, tuning materials to improve performance for OER catalysis also impacts their performance for MOR catalysis.

## Methods and Materials

### Nanoparticle Synthesis

We used a borosilicate round-bottom flask with three necks as a synthesis vessel. Prior to each synthesis, we soaked the flask overnight in an acid bath of 10% HNO_3_ by volume and then rinsed it with copious amounts of deionized H_2_O. To synthesize Fe(0) cores, we combined 4 mL of an aqueous solution of 25 g/L iron(II) sulfate heptahydrate with the ligand stabilizer amino tris(methylene phosphonic acid) (ATMP) at a ratio of 0.05 mol ATMP: mol Fe. We prepared all aqueous solutions under ambient conditions with deionized H_2_O that we sparged with Ar to remove dissolved O_2_. We added H_2_O so that the final volume after all additions was 21 mL. Therefore, the H_2_O present during the Fe(0) core formation decreased by 11% over the synthesis series. We bubbled the ATMP-stabilized Fe solution with Ar for 15 min while mixing the solution on an orbital shaker to remove dissolved O_2_ remaining in solution. We prepared a fresh solution of sodium borohydride (NaBH_4_) in H_2_O and added it dropwise by syringe while mixing by hand to reduce the stabilized Fe(II) to Fe(0) nanoparticles. The ratio of 2.2 mol BH_4_^−^: mol Fe provides 2 mol of BH_4_^−^ per 1 mol of Fe for complete reduction of all Fe(II) atoms, with an excess 10% to accommodate the side reaction of BH_4_^−^ with H_2_O^25^. We mixed the suspension with an orbital shaker for 30 min to allow for complete Fe(II) reduction while holding the flask under reduced pressure to draw out evolving H_2_ gas. ATMP-stabilized Fe(0) nanoparticles synthesized in this way have a primary nanoparticle diameter from approximately 100 nm to approximately 200 nm based on scanning electron microscopy and transmission electron microscopy^26^. Ni(II) adsorption and displacement of Fe(0) created the Ni-rich shell. We combined an aqueous solution of 100 g/L nickel(II) chloride hexahydrate with the stabilizer polyvinylpyrrolidone (PVP) with a molecular weight of 40 000 (PVP_40_) at a ratio of 0.00125 mol PVP_40_: mol Ni. After releasing the vacuum on the flask, we added the PVP_40_-stabilized Ni solution dropwise by syringe to the Fe(0) nanoparticle suspension while mixing by hand. We then mixed the suspension on an orbital shaker for 15 min while holding the flask under reduced pressure. Ni/Fe nanoparticles formed with a molar ratio of 1 mol Ni:1 mol Fe had a primary nanoparticle diameter from approximately 50 nm to approximately 150 nm based on scanning transmission electron microscopy^16^. Energy dispersive X-ray spectroscopy composition maps showed that dense metallic nanoparticles had a core-shell structure with an Fe-rich core and a Ni-rich shell, although both elements were present throughout the nanoparticle volume^16^. We tested seven molar ratios of Ni:Fe in the synthesis vessel: 0.2 mol Ni:1 mol Fe, 0.5 mol Ni:1 mol Fe, 0.8 mol Ni:1 mol Fe, 1 mol Ni:1 mol Fe, 1.2 mol Ni:1 mol Fe, 1.5 mol Ni:1 mol Fe, and 2 mol Ni:1 mol Fe. We centrifuged the resulting suspension of Ni/Fe nanoparticles at 4225 relative centrifugal force (RCF) for 1 min to pellet the nanoparticles and removed the supernatant, containing unreacted metal salts and excess stabilizer, by pipette. We resuspended the isolated Ni/Fe nanoparticles in methanol (CH_3_OH)^27^ to produce a nominal concentration of 2 g/L, based on complete reduction of Fe.

### Electrochemical Measurements

Prior to each electrochemical experiment, we cleaned the glassy carbon electrode by gently polishing the surface in an alumina slurry and then rinsed it with copious amounts of deionized H_2_O. We verified that the electrode was clean by the absence of O_2_ evolution, as we describe below. We prepared catalyst inks by combining equal volumes of a 2 g/L suspension of Ni/Fe nanoparticles in CH_3_OH, and a solution of alkaline exchange-membrane ionomer^16^ in CH_3_OH with a mass percent of 0.035% to give a mass ratio of 6:1. We mixed the ink by sonication in an ice-water bath for at least 10 min. Immediately after sonication, we drop-cast 2 µL of ink on a clean electrode of glassy carbon with a surface area of 0.07 cm^2^ and dried the ink in air at room temperature. The nominal mass of nanoparticles on the electrode was 2 µg. We measured electrocatalytic performance of the nanoparticles for O_2_ evolution and CH_3_OH oxidation using a three-electrode cell with Ag/AgCl in 3 mol/L KCl as the reference electrode and a graphite rod as the counter electrode. Prior to the measurements, we bubbled the 1 mol/L NaOH electrolyte with N_2_ for 30 min to remove dissolved O_2_, and then continuously flowed N_2_ through the headspace above the electrolyte. We placed the Ag/AgCl reference electrode in a salt bridge containing 3 mol/L NaCl for all experiments to prevent Ag_2_O formation from NaOH exposure. We performed cyclic voltammetry (CV) with a voltage window of 0.0 V to 0.8 V *versus* Ag/AgCl. We measured O_2_ evolution in 1 mol/L NaOH by cycling the samples 30 times. Subsequently, we measured CH_3_OH oxidation by adding 0.2 mol/L, 1 mol/L, and 2 mol/L CH_3_OH to the NaOH electrolyte, cycling the samples 10 times at each concentration. The scan rate was 20 mV/s for all measurements. We converted *E*_*MEAS*_, the measured potential versus Ag/AgCl, to *E*_*RHE*_, the potential versus the reversible hydrogen electrode (RHE), using the equation, *E*_*RHE*_ = *E*_*MEAS*_ + *0.059*·*pH* + *E*^*0*^_*Ag/AgCl*_, where *pH* is 14.0 for the 1 mol/L NaOH electrolyte and *E*^*0*^_*Ag/AgCl*_ is 0.21 V for the Ag/AgCl reference electrode in 3 mol/L NaCl. We adjusted all electrochemical data using a correction for *iR*_*u*_, where *i* is the current and *R*_*u*_ is the uncompensated series resistance. We determined *R*_*u*_ by potentiostatic electrochemical impedance spectroscopy. We subtracted the calculated value of *iR*_*u*_ from the measured potential versus RHE for all CV measurements. The overpotential (*η*) to achieve a current density of 10 mA/cm^2^ (geometric area) is a useful benchmark for OER catalysts because this is the approximate current density required for a device converting solar energy to fuel with 10% efficiency^15^. We determined this value by subtracting the reversible potential of the oxygen evolution reaction (*E*_*REV*_), *η* = *E*_*RHE*_ - *E*_*REV*_, where *E*_*REV*_ = 1.23 V.

### Inductively Coupled Plasma-Atomic Emission Spectroscopy (ICP-AES)

We purified nanoparticle samples in CH_3_OH for elemental analysis by centrifuging aliquots of approximately 250 µL at 18 500 RCF for 1 min to pellet the nanoparticles, removing the CH_3_OH, and dissolving the particles in an aqueous solution of HNO_3_ with a volume fraction of 5%, resulting in a final volume of 10 mL. We analyzed a minimum of five aliquots for each synthesis. The ICP-AES instrument had a detection limit for Ni of approximately 0.02 µg/mL and a detection limit for Fe of approximately 0.05 µg/mL. Our samples had an Fe content three orders of magnitude greater than the detection limit and a Ni content nearly an order of magnitude greater than the detection limit, for the sample with the smallest amount of Ni. We converted measured Ni and Fe concentrations to bulk nanoparticle composition by accounting for the mass of each aliquot.

### X-ray Photoelectron Spectroscopy (XPS)

We prepared samples of purified nanoparticles in CH_3_OH for surface analysis by drop-casting aliquots onto clean silicon wafers, drying them in air, and storing them under N_2_ until transfer to vacuum. We used the low-energy electron flood gun of the XPS instrument for charge neutralization and excited our samples with a monochromatic Al Kα X-ray source. We measured electrons with a take-off angle of 90° perpendicular to the sample surface, resulting in a probe depth of approximately 10 nm. Each analysis region had a diameter of approximately 500 µm. We adjusted the binding energy scale of each spectrum to the C 1s C-C/C-H signal, which we set to 284.5 eV. We obtained survey spectra with a pass energy of 160 eV, 1 eV per step, and a dwell time of 3 s per step to determine the relative elemental composition for each sample. We obtained high energy resolution spectra with a pass energy of 40 eV, 0.1 eV per step, and a dwell time of 3 s per step for the C 1 s, O 1 s, Fe 2p, and Ni 2p regions to provide information on Ni and Fe bonding states.

### Data availability

All data are available from the corresponding author on reasonable request.

## Electronic supplementary material

Supplementary Information

## References

[1] Cho S-J, Jarrett BR, Louie AY, Kauzlarich SM (2006). Gold-coated iron nanoparticles: a novel magnetic resonance agent for T_1_ and T_2_ weighted imaging. Nanotechnol..

[2] Smolensky ED, Neary MC, Zhou Y, Berquo TS, Pierre VC (2011). Fe_3_O_4_@organic@Au: core-shell nanocomposites with high saturation magnetisation as magnetopasmonic MRI contrast agents. Chem. Commun..

[3] Yuan Q (2014). Performance of nano-nickel core wrapped with Pt crystalline thin film for methanol electro-oxidation. J. Power Sources.

[4] Tschulik K (2015). Core-shell nanoparticles: characterizating multifunctional materials beyond imaging-distinguishing and quantifying perfect and broken shells. Adv. Funct. Mater..

[5] Li X, Walsh FC, Pletcher D (2011). Nickel based electrocatalysts for oxygen evolution in high current density alklaine water electrolysers. Phys. Chem. Chem. Phys..

[6] Gong M, Dai H (2015). A mini review of NiFe-based materials as highly active oxygen evolution reaction electrocatalysts. Nano Research.

[7] Trotochaud L, Ranney JK, Williams KN, Boettcher SW (2012). Solution-cast metal oxide thin film electrocatalysts for oxygen evolution. J. Am. Chem. Soc..

[8] Trotochaud L, Young SL, Ranney JK, Boettcher SW (2014). Nickel-iron oxyhydroxide oxygen-evolution electrocatalysts: the role of intentional and incidental iron incorporation. J. Am. Chem. Soc..

[9] Smith RDL, Prevot MS, Fagan RD, Trudel S, Berlinguette CP (2013). Water oxidation catalysis: electrocatalytic response to metal stoichiometry in amorphous metal oxide films containing iron, cobalt, and nickel. J. Am. Chem. Soc..

[10] Louie MW, Bell AT (2013). An investigation of thin-film Ni-Fe oxide catalysts for the electrochemical evolution of oxygen. J. Am. Chem. Soc..

[11] Li X-q, Zhang W-x (2006). Iron nanoparticles: the core-shell structure and unique properties for Ni(II) sequestration. Langmuir.

[12] Vanysek, P. Electrochemical series in *CRC handbook of chemistry and physics* (ed. Haynes, W. M.) 78–84 (CRC Press, 2017).

[13] Li X-q, Zhang W-x (2007). Sequestration of metal cations with zerovalent iron nanoparticles - a study with high resolution X-ray photoelectron spectroscopy. J. Phys. Chem. C.

[14] Yin, H. *et al*. Remarkably enhanced water splitting activity of nickel foam due to simple immersion in a ferric nitrate solution. *Nano Res*. 10.1007/s12274-017-1886-7 (2017).

[15] McCrory CCL, Jung S, Peters JC, Jaramillo TF (2013). Benchmarking heterogeneous electrocatalysts for the oxygen evolution reaction. J. Am. Chem. Soc..

[16] Candelaria SL (2017). Multi-component Fe-Ni hydroxide nanocatalyst for oxygen evolution and methanol oxidation reactions under alkaline conditions. ACS Catal..

[17] Skowronski JM, Wazny A (2006). Nickel foam-based Ni(OH)_2_/NiOOH electrode as catalytic system for methanol oxidation in alkaline solution. J. New Mat. Electrochem. Systems.

[18] Fairley, N. & Carrick, A. The casa cookbook part 1: recipes for XPS data processing (AcolyteScience, 2005).

[19] Harriman A, Pickering IJ, Thomas JM, Christensen PA (1984). Metal oxides as heterogeneous catalysts for oxygen evolution under photochemical conditions. J. Chem. Soc. Faraday Trans..

[20] Biesinger MC (2011). Resolving surface chemical states in XPS analysis of first row transition metals, oxides, and hydroxides: Cr, Mn, Fe, Co, and Ni. Appl. Surf. Sci..

[21] Grosvenor AP, Kobe BA, Biesinger MC, McIntyre NS (2004). Investigation of multiplet splitting of Fe 2p XPS spectra and bonding in iron compounds. Surf. Interface Anal..

[22] Yamashita T, Hayes P (2008). Analysis of XPS spectra of Fe^2+^ and Fe^3+^ ions in oxide materials. Appl. Surf. Sci..

[23] Shirley DA (1972). High resolution X-ray photoemission spectrum of the valence bands of gold. Phys. Rev. B.

[24] Grosvenor AP (2006). New interpretations of XPS spectra of nickel metal and oxides. Surf. Sci..

[25] Lo C-tF, Karan K, Davis BR (2007). Kinetic studies of reaction between sodium borohydride and methanol, water, and their mixtures. Ind. Eng. Chem. Res..

[26] Greenlee LF, Rentz NS (2014). ATMP-stabilized iron nanoparticles: chelator-controlled nanoparticle synthesis. J. Nano. Res..

[27] Sriram I (2015). Stability and phase transfer of catalytically active platinum nanoparticle suspensions. J. Nano. Res..

