# Supplementary material for: Rapid Synthesis and Correlative Measurements of Electrocatalytic Nickel/Iron Oxide Nanoparticles
Source: Sci Rep. 2018 Mar 15;8:4584. doi: 10.1038/s41598-018-22609-x (PMC5854608; doi:10.1038/s41598-018-22609-x)
Supplement: Supplementary file 1 — Supplementary Information [file 41598_2018_22609_MOESM1_ESM.pdf]

Supplementary Information

Rapid Synthesis and Correlative Measurements of Electrocatalytic Nickel/Iron Oxide Nanoparticles

Kavita M. Jeerage[1,*], Stephanie L. Candelaria[1], and Samuel M. Stavis[2]

[1]Applied Chemicals and Materials Division, National Institute of Standards and Technology, 325 Broadway, Boulder, Colorado 80305, United States

[2]Center for Nanoscale Science and Technology, National Institute of Standards and Technology, 100 Bureau Drive, Gaithersburg, Maryland 20899, United States

*Address correspondence to jeerage@boulder.nist.gov

We examined the structure and composition of individual Ni/Fe nanoparticles by scanning transmission electron microscopy (STEM) and energy-dispersive X-ray spectroscopy (EDS). Electron micrographs and elemental maps of representative nanoparticles show that our synthesis process results in nanoparticles with multiple structures and compositions. We first present Ni/Fe nanoparticles with the expected core-shell structure (Figure S1). The brightfield micrograph shows multiple nanoparticles that are clearly visible in the Fe elemental map, indicating three Fe cores. The large central nanoparticle is also discernable in the Ni elemental map because the Ni distribution is slightly greater at the nanoparticle edges. This is where the electron beam primarily travels through the Ni shell, instead of interacting with both the Ni shell and the Fe core. We next present Ni-rich organic nanoparticles (Figure S2). The nanoparticle cluster in the brightfield micrograph is not clear in the Fe elemental map, but is clearly visible in the Ni and P elemental maps. These nanoparticles were easily damaged by the electron beam, evidence for organic material. In STEM images in which individual nanoparticles can be distinguished, Ni/Fe core-shell nanoparticles appear darker, indicating the greater electron-stopping power of dense cores. Ni-rich organic particles appear lighter, indicating low-density cores. Based on this rubric, the dark particle just below the cluster of Ni-rich organic particles (Figure S2) would be expected to have a Ni/Fe core-shell structure and this was confirmed by elemental analysis (not shown). However, this is not a reliable classification method because it only allows the evaluation of individual nanoparticles that do not overlay other nanoparticles.

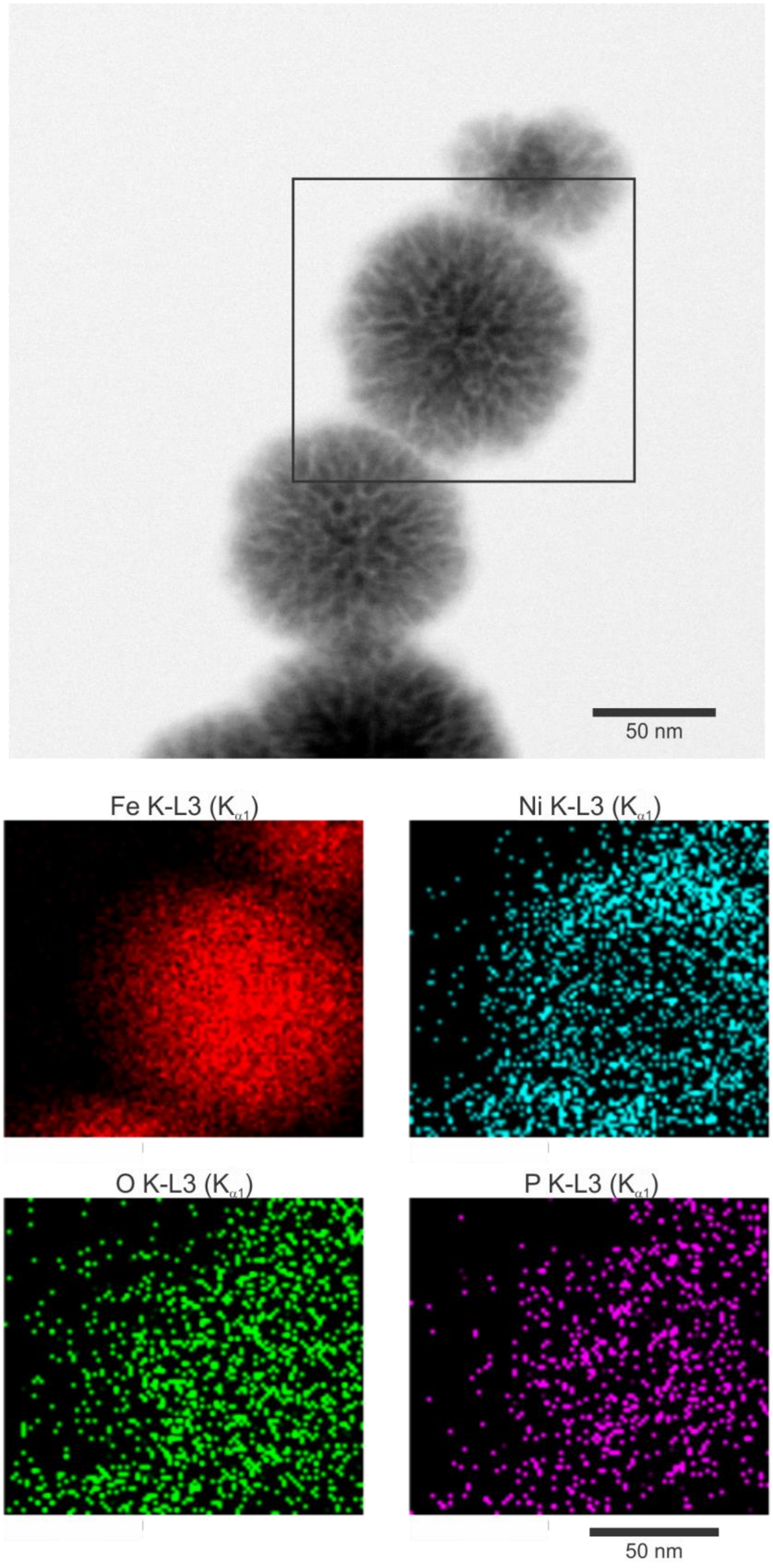

Figure S1. Brightfield STEM image (top) and corresponding EDS elemental maps for Fe, Ni, O, and P. Rectangle indicates the region of elemental analysis.

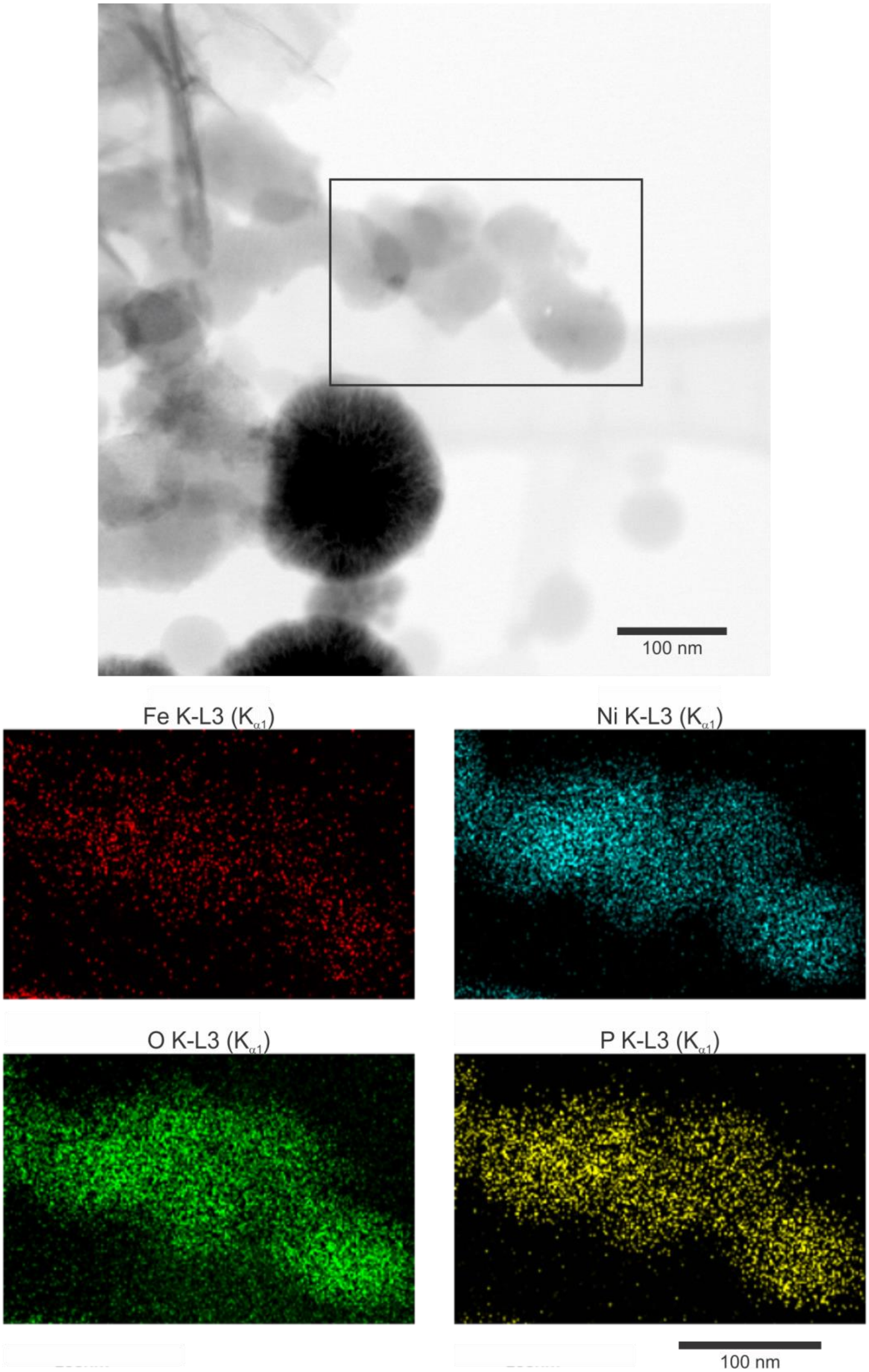

Figure S2. Brightfield STEM image (top) and corresponding EDS elemental maps for Fe, Ni, O, and P. Rectangle indicates the region of elemental analysis.

Methods and Materials

We prepared samples by diluting purified nanoparticles (2 g/Lin CH_3OH) by a factor of 50 in CH_3OH. We dropcast 3 μL aliquots of the dilute nanoparticle suspension onto TEM grids consisting of ultrathin carbon supported by lacey carbon and allowed the CH_3OH to evaporate. We prepared samples and transferred them into the vacuum chamber within 4 h to limit nanoparticle oxidation. We also cleaned the samples in argon plasma for 45 s immediately prior to transfer. We used a STEM system with spherical-aberration correction and operated it at an accelerating voltage of 200 kV. We show all electron micrographs as brightfield images. We acquired electron micrographs with an image size of 1024 pixels by 1024 pixels, however, the images shown here were cropped slightly around the region of interest. We performed EDS in STEM mode with a silicon drift detector.
